# Supplementary material for: Repetitive Transcranial Magnetic Stimulation for Tobacco Treatment in Cancer Patients: A Preliminary Report of a One-Week Treatment
Source: J Smok Cessat. 2022 Jul 11;2022:2617146. doi: 10.1155/2022/2617146 (PMC9293555; doi:10.1155/2022/2617146)
Supplement: Supplementary Materials — Mixed-Models for Repeated Measures Analysis: (1) self-reported cigarette consumption, (2) Questionnaire of Smoking Urges-Brief (QSU-B), and (3) carbon monoxide levels. [file 2617146.f1.docx]

**Supplementary Material**

**Mixed-Models for Repeated Measures Analysis**

**Note: The original data is available upon request from the corresponding author**.

1. Self-reported Cigarette Consumption

Note: The “CPDdata2” dataset is in long format, with a baseline measure and measurement during each of 5 rTMS sessions.

SAS programming code:

/* full model */

proc mixed data= CPDdata2;

class subject_ visit_num TrGroup;

model CigarettePerday = TrGroup visit_num TrGroup*visit_num CigarettePerday_bl / solution;

repeated / subject=subject_ type=ar(1) rcorr;

run;

/* null model - for aid in calculation of f squared statistic */

proc mixed data= CPDdata2;

class subject_ visit_num TrGroup;

model CigarettePerday = visit_num CigarettePerday_bl;

repeated / subject=subject_ type=ar(1) rcorr;

run;

Output from model estimation for the “full model”:

The Mixed Procedure

Model Information

Data Set WORK.CPDDATA2

Dependent Variable CigarettePerday

Covariance Structure Autoregressive

Subject Effect Subject_

Estimation Method REML

Residual Variance Method Profile

Fixed Effects SE Method Model-Based

Degrees of Freedom Method Between-Within

Class Level Information

Class Levels Values

Subject_ 7 10002 10003 10004 10005 20001

20002 20003

visit_num 6 0 1 2 3 4 5

TrGroup 2 X Y

Dimensions

Covariance Parameters 2

Columns in X 22

Columns in Z 0

Subjects 7

Max Obs per Subject 6

Number of Observations

Number of Observations Read 42

Number of Observations Used 42

Number of Observations Not Used 0

Iteration History

Iteration Evaluations -2 Res Log Like Criterion

0 1 181.14762353

1 2 173.52687218 0.00000180

2 1 173.52676361 0.00000000

Convergence criteria met.

The Mixed Procedure

Estimated R Correlation Matrix for Subject_ 10002

Row Col1 Col2 Col3 Col4 Col5 Col6

1 1.0000 0.4989 0.2489 0.1242 0.06196 0.03091

2 0.4989 1.0000 0.4989 0.2489 0.1242 0.06196

3 0.2489 0.4989 1.0000 0.4989 0.2489 0.1242

4 0.1242 0.2489 0.4989 1.0000 0.4989 0.2489

5 0.06196 0.1242 0.2489 0.4989 1.0000 0.4989

6 0.03091 0.06196 0.1242 0.2489 0.4989 1.0000

Covariance Parameter Estimates

Cov Parm Subject Estimate

AR(1) Subject_ 0.4989

Residual 14.0068

Fit Statistics

-2 Res Log Likelihood 173.5

AIC (Smaller is Better) 177.5

AICC (Smaller is Better) 178.0

BIC (Smaller is Better) 177.4

Null Model Likelihood Ratio Test

DF Chi-Square Pr > ChiSq

1 7.62 0.0058

Solution for Fixed Effects

Standard

Effect X = sham, Y = active visit_num Estimate Error DF t Value

Intercept -2.6318 2.9386 4 -0.90

TrGroup X 4.4652 2.9178 4 1.53

Solution for Fixed Effects

Effect X = sham, Y = active visit_num Pr > |t|

Intercept 0.4211

TrGroup X 0.2007

The Mixed Procedure

Solution for Fixed Effects

Standard

Effect X = sham, Y = active visit_num Estimate Error DF t Value

TrGroup Y 0 . . .

visit_num 0 7.0000 3.0082 25 2.33

visit_num 1 6.3333 2.9596 25 2.14

visit_num 2 -0.3333 2.8598 25 -0.12

visit_num 3 -0.6667 2.6483 25 -0.25

visit_num 4 0.6667 2.1631 25 0.31

visit_num 5 0 . . .

visit_num*TrGroup X 0 -5.7500 3.9795 25 -1.44

visit_num*TrGroup Y 0 0 . . .

visit_num*TrGroup X 1 -5.8333 3.9152 25 -1.49

visit_num*TrGroup Y 1 0 . . .

visit_num*TrGroup X 2 1.5833 3.7831 25 0.42

visit_num*TrGroup Y 2 0 . . .

visit_num*TrGroup X 3 0.6667 3.5034 25 0.19

visit_num*TrGroup Y 3 0 . . .

visit_num*TrGroup X 4 -0.4167 2.8615 25 -0.15

visit_num*TrGroup Y 4 0 . . .

visit_num*TrGroup X 5 0 . . .

visit_num*TrGroup Y 5 0 . . .

CigarettePerday_bl 0.7430 0.1172 4 6.34

Solution for Fixed Effects

Effect X = sham, Y = active visit_num Pr > |t|

TrGroup Y .

visit_num 0 0.0284

visit_num 1 0.0423

visit_num 2 0.9081

visit_num 3 0.8033

visit_num 4 0.7605

visit_num 5 .

visit_num*TrGroup X 0 0.1609

visit_num*TrGroup Y 0 .

visit_num*TrGroup X 1 0.1488

visit_num*TrGroup Y 1 .

visit_num*TrGroup X 2 0.6791

visit_num*TrGroup Y 2 .

visit_num*TrGroup X 3 0.8506

visit_num*TrGroup Y 3 .

visit_num*TrGroup X 4 0.8854

visit_num*TrGroup Y 4 .

visit_num*TrGroup X 5 .

visit_num*TrGroup Y 5 .

CigarettePerday_bl 0.0032

The Mixed Procedure

Type 3 Tests of Fixed Effects

Num Den

Effect DF DF F Value Pr > F

TrGroup 1 4 2.29 0.2051

visit_num 5 25 1.60 0.1980

visit_num*TrGroup 5 25 1.53 0.2168

CigarettePerday_bl 1 4 40.23 0.0032

Output from model estimation for the “null model”:

The Mixed Procedure

Model Information

Data Set WORK.CPDDATA2

Dependent Variable CigarettePerday

Covariance Structure Autoregressive

Subject Effect Subject_

Estimation Method REML

Residual Variance Method Profile

Fixed Effects SE Method Model-Based

Degrees of Freedom Method Between-Within

Class Level Information

Class Levels Values

Subject_ 7 10002 10003 10004 10005 20001

20002 20003

visit_num 6 0 1 2 3 4 5

TrGroup 2 X Y

Dimensions

Covariance Parameters 2

Columns in X 8

Columns in Z 0

Subjects 7

Max Obs per Subject 6

Number of Observations

Number of Observations Read 42

Number of Observations Used 42

Number of Observations Not Used 0

Iteration History

Iteration Evaluations -2 Res Log Like Criterion

0 1 216.06796912

1 2 205.11045832 0.00000263

2 1 205.11027324 0.00000000

Convergence criteria met.

The Mixed Procedure

Estimated R Correlation Matrix for Subject_ 10002

Row Col1 Col2 Col3 Col4 Col5 Col6

1 1.0000 0.5394 0.2910 0.1570 0.08468 0.04568

2 0.5394 1.0000 0.5394 0.2910 0.1570 0.08468

3 0.2910 0.5394 1.0000 0.5394 0.2910 0.1570

4 0.1570 0.2910 0.5394 1.0000 0.5394 0.2910

5 0.08468 0.1570 0.2910 0.5394 1.0000 0.5394

6 0.04568 0.08468 0.1570 0.2910 0.5394 1.0000

Covariance Parameter Estimates

Cov Parm Subject Estimate

AR(1) Subject_ 0.5394

Residual 16.1927

Fit Statistics

-2 Res Log Likelihood 205.1

AIC (Smaller is Better) 209.1

AICC (Smaller is Better) 209.5

BIC (Smaller is Better) 209.0

Null Model Likelihood Ratio Test

DF Chi-Square Pr > ChiSq

1 10.96 0.0009

Type 3 Tests of Fixed Effects

Num Den

Effect DF DF F Value Pr > F

visit_num 5 30 1.11 0.3769

CigarettePerday_bl 1 5 31.69 0.0025

1. Questionnaire of Smoking Urges-Brief (QSU-B)

Note: The “QSUdata2” dataset is in long format, with a baseline measure and 2 measurements during each of 5 rTMS sessions (1 pre-session and 1 post-session).

/* full model – the AIC from this model was 408.3, indicating that this model with a compound symmetry error structure provided a better fit than the model with an AR(1) structure, which had an AIC of 416.4 */

proc mixed data=QSUdata2;

class subjectID visitNumber TrGroup prepost;

model qsu_score = TrGroup visitNumber TrGroup*visitNumber prepost TrGroup*prepost visitNumber*prepost TrGroup*visitNumber*prepost qsu_baseline;

repeated / subject=subjectID type=cs rcorr;

run;

/* null model - for aid in calculation of f squared statistic */

proc mixed data=QSUdata2;

class subjectID visitNumber TrGroup prepost;

model qsu_score = visitNumber prepost qsu_baseline;

repeated / subject=subjectID type=cs rcorr;

run;

Output from model estimation for the “full model”:

The Mixed Procedure

Model Information

Data Set WORK.QSUDATA2

Dependent Variable qsu_score

Covariance Structure Compound Symmetry

Subject Effect subjectID

Estimation Method REML

Residual Variance Method Profile

Fixed Effects SE Method Model-Based

Degrees of Freedom Method Between-Within

Class Level Information

Class Levels Values

subjectID 7 10002 10003 10004 10005 20001

20002 20003

visitNumber 5 1.00 2.00 3.00 4.00 5.00

TrGroup 2 X Y

PrePost 2 POST PRE

Dimensions

Covariance Parameters 2

Columns in X 55

Columns in Z 0

Subjects 7

Max Obs per Subject 10

Number of Observations

Number of Observations Read 70

Number of Observations Used 70

Number of Observations Not Used 0

Iteration History

Iteration Evaluations -2 Res Log Like Criterion

0 1 422.20410376

1 1 404.34306758 0.00000000

Convergence criteria met.

Estimated R Correlation Matrix for subjectID 10002

Row Col1 Col2 Col3 Col4 Col5 Col6 Col7 Col8 Col9

1 1.0000 0.4749 0.4749 0.4749 0.4749 0.4749 0.4749 0.4749 0.4749

2 0.4749 1.0000 0.4749 0.4749 0.4749 0.4749 0.4749 0.4749 0.4749

3 0.4749 0.4749 1.0000 0.4749 0.4749 0.4749 0.4749 0.4749 0.4749

4 0.4749 0.4749 0.4749 1.0000 0.4749 0.4749 0.4749 0.4749 0.4749

5 0.4749 0.4749 0.4749 0.4749 1.0000 0.4749 0.4749 0.4749 0.4749

6 0.4749 0.4749 0.4749 0.4749 0.4749 1.0000 0.4749 0.4749 0.4749

7 0.4749 0.4749 0.4749 0.4749 0.4749 0.4749 1.0000 0.4749 0.4749

8 0.4749 0.4749 0.4749 0.4749 0.4749 0.4749 0.4749 1.0000 0.4749

9 0.4749 0.4749 0.4749 0.4749 0.4749 0.4749 0.4749 0.4749 1.0000

10 0.4749 0.4749 0.4749 0.4749 0.4749 0.4749 0.4749 0.4749 0.4749

Estimated R

Correlation

Matrix for

subjectID

10002

Row Col10

1 0.4749

2 0.4749

3 0.4749

4 0.4749

5 0.4749

6 0.4749

7 0.4749

8 0.4749

9 0.4749

10 1.0000

Covariance Parameter Estimates

Cov Parm Subject Estimate

CS subjectID 80.6655

Residual 89.2085

Fit Statistics

-2 Res Log Likelihood 404.3

AIC (Smaller is Better) 408.3

AICC (Smaller is Better) 408.6

BIC (Smaller is Better) 408.2

Null Model Likelihood Ratio Test

DF Chi-Square Pr > ChiSq

1 17.86 <.0001

Type 3 Tests of Fixed Effects

Num Den

Effect DF DF F Value Pr > F

TrGroup 1 4 0.01 0.9382

visitNumber 4 20 3.99 0.0154

visitNumber*TrGroup 4 20 6.96 0.0011

PrePost 1 5 0.39 0.5601

TrGroup*PrePost 1 5 2.44 0.1791

visitNumber*PrePost 4 20 0.88 0.4910

visitN*TrGrou*PrePos 4 20 0.80 0.5379

qsu_baseline 1 4 61.62 0.0014

Output from model estimation for the “null model”:

The Mixed Procedure

Model Information

Data Set WORK.QSUDATA2

Dependent Variable qsu_score

Covariance Structure Compound Symmetry

Subject Effect subjectID

Estimation Method REML

Residual Variance Method Profile

Fixed Effects SE Method Model-Based

Degrees of Freedom Method Between-Within

Class Level Information

Class Levels Values

subjectID 7 10002 10003 10004 10005 20001

20002 20003

visitNumber 5 1.00 2.00 3.00 4.00 5.00

TrGroup 2 X Y

PrePost 2 POST PRE

Dimensions

Covariance Parameters 2

Columns in X 9

Columns in Z 0

Subjects 7

Max Obs per Subject 10

Number of Observations

Number of Observations Read 70

Number of Observations Used 70

Number of Observations Not Used 0

Iteration History

Iteration Evaluations -2 Res Log Like Criterion

0 1 530.41496981

1 1 519.05777415 0.00000000

Convergence criteria met.

Estimated R Correlation Matrix for subjectID 10002

Row Col1 Col2 Col3 Col4 Col5 Col6 Col7 Col8 Col9

1 1.0000 0.3212 0.3212 0.3212 0.3212 0.3212 0.3212 0.3212 0.3212

2 0.3212 1.0000 0.3212 0.3212 0.3212 0.3212 0.3212 0.3212 0.3212

3 0.3212 0.3212 1.0000 0.3212 0.3212 0.3212 0.3212 0.3212 0.3212

4 0.3212 0.3212 0.3212 1.0000 0.3212 0.3212 0.3212 0.3212 0.3212

5 0.3212 0.3212 0.3212 0.3212 1.0000 0.3212 0.3212 0.3212 0.3212

6 0.3212 0.3212 0.3212 0.3212 0.3212 1.0000 0.3212 0.3212 0.3212

7 0.3212 0.3212 0.3212 0.3212 0.3212 0.3212 1.0000 0.3212 0.3212

8 0.3212 0.3212 0.3212 0.3212 0.3212 0.3212 0.3212 1.0000 0.3212

9 0.3212 0.3212 0.3212 0.3212 0.3212 0.3212 0.3212 0.3212 1.0000

10 0.3212 0.3212 0.3212 0.3212 0.3212 0.3212 0.3212 0.3212 0.3212

Estimated R

Correlation

Matrix for

subjectID

10002

Row Col10

1 0.3212

2 0.3212

3 0.3212

4 0.3212

5 0.3212

6 0.3212

7 0.3212

8 0.3212

9 0.3212

10 1.0000

Covariance Parameter Estimates

Cov Parm Subject Estimate

CS subjectID 59.2661

Residual 125.25

Fit Statistics

-2 Res Log Likelihood 519.1

AIC (Smaller is Better) 523.1

AICC (Smaller is Better) 523.3

BIC (Smaller is Better) 522.9

Null Model Likelihood Ratio Test

DF Chi-Square Pr > ChiSq

1 11.36 0.0008

Type 3 Tests of Fixed Effects

Num Den

Effect DF DF F Value Pr > F

visitNumber 4 24 2.02 0.1243

PrePost 1 6 0.52 0.4974

qsu_baseline 1 5 78.78 0.0003

1. Carbon Monoxide Levels

Note: The “CO2” dataset is in long format, with a carbon monoxide measurement during each of 5 rTMS sessions.

SAS programming code:

/* full model – note that the model using an AR(1) covariance structure did not converge and thus the compound symmetry structure was used. */

proc mixed data=co2;

class subjectidco visit_num TrGroupco ;

model co = TrGroupco visit_num TrGroupco*visit_num co_bl;

repeated int / subject=subjectidco type=cs rcorr;

run;

/* null model - for aid in calculation of f squared statistic */

proc mixed data=co2 ;

class subjectidco visit_num ;

model co = visit_num co_bl;

repeated int / subject=subjectidco type=cs r;

run;

Output from model estimation for the “full model”:

The Mixed Procedure

Model Information

Data Set WORK.CO2

Dependent Variable CO

Covariance Structure Compound Symmetry

Subject Effect subjectIDCO

Estimation Method REML

Residual Variance Method Profile

Fixed Effects SE Method Model-Based

Degrees of Freedom Method Between-Within

Class Level Information

Class Levels Values

subjectIDCO 7 10002 10003 10004 10005 20001

20002 20003

visit_num 5 TMS1 TMS2 TMS3 TMS4 TMS5

TrGroupCO 2 active sham

Dimensions

Covariance Parameters 2

Columns in X 19

Columns in Z 0

Subjects 7

Max Obs per Subject 5

Number of Observations

Number of Observations Read 35

Number of Observations Used 35

Number of Observations Not Used 0

Iteration History

Iteration Evaluations -2 Res Log Like Criterion

0 1 137.66424417

1 1 136.37047204 0.00000000

Convergence criteria met.

Estimated R Correlation Matrix for subjectIDCO 10002

Row Col1 Col2 Col3 Col4 Col5

1 1.0000 0.1996 0.1996 0.1996 0.1996

2 0.1996 1.0000 0.1996 0.1996 0.1996

3 0.1996 0.1996 1.0000 0.1996 0.1996

4 0.1996 0.1996 0.1996 1.0000 0.1996

5 0.1996 0.1996 0.1996 0.1996 1.0000

Covariance Parameter Estimates

Cov Parm Subject Estimate

CS subjectIDCO 1.6533

Residual 6.6283

Fit Statistics

-2 Res Log Likelihood 136.4

AIC (Smaller is Better) 140.4

AICC (Smaller is Better) 140.9

BIC (Smaller is Better) 140.3

Null Model Likelihood Ratio Test

DF Chi-Square Pr > ChiSq

1 1.29 0.2554

Type 3 Tests of Fixed Effects

Num Den

Effect DF DF F Value Pr > F

TrGroupCO 1 4 0.27 0.6300

visit_num 4 20 1.04 0.4128

visit_num*TrGroupCO 4 20 1.14 0.3680

co_bl 1 4 111.07 0.0005

Output from model estimation for the “null model”:

The SAS System 08:43 Wednesday, January 26, 2022 59

The Mixed Procedure

Model Information

Data Set WORK.CO2

Dependent Variable CO

Covariance Structure Compound Symmetry

Subject Effect subjectIDCO

Estimation Method REML

Residual Variance Method Profile

Fixed Effects SE Method Model-Based

Degrees of Freedom Method Between-Within

Class Level Information

Class Levels Values

subjectIDCO 7 10002 10003 10004 10005 20001

20002 20003

visit_num 5 TMS1 TMS2 TMS3 TMS4 TMS5

Dimensions

Covariance Parameters 2

Columns in X 7

Columns in Z 0

Subjects 7

Max Obs per Subject 5

Number of Observations

Number of Observations Read 35

Number of Observations Used 35

Number of Observations Not Used 0

Iteration History

Iteration Evaluations -2 Res Log Like Criterion

0 1 158.93382303

1 1 157.99852770 0.00000000

Convergence criteria met.

Estimated R Correlation Matrix for subjectIDCO 10002

Row Col1 Col2 Col3 Col4 Col5

1 1.0000 0.1492 0.1492 0.1492 0.1492

2 0.1492 1.0000 0.1492 0.1492 0.1492

3 0.1492 0.1492 1.0000 0.1492 0.1492

4 0.1492 0.1492 0.1492 1.0000 0.1492

5 0.1492 0.1492 0.1492 0.1492 1.0000

Covariance Parameter Estimates

Cov Parm Subject Estimate

CS subjectIDCO 1.1891

Residual 6.7786

Fit Statistics

-2 Res Log Likelihood 158.0

AIC (Smaller is Better) 162.0

AICC (Smaller is Better) 162.5

BIC (Smaller is Better) 161.9

Null Model Likelihood Ratio Test

DF Chi-Square Pr > ChiSq

1 0.94 0.3335

Type 3 Tests of Fixed Effects

Num Den

Effect DF DF F Value Pr > F

visit_num 4 24 1.11 0.3745

co_bl 1 5 141.40 <.0001
